# Numerical Simulation of Natural Convection of a Nanofluid in an Inclined Heated Enclosure Using Two-Phase Lattice Boltzmann Method: Accurate Effects of Thermophoresis and Brownian Forces

**DOI:** 10.1186/s11671-015-1006-0

**Published:** 2015-07-16

**Authors:** Mahmoud Ahmed, Morteza Eslamian

**Affiliations:** Department of Mechanical Engineering, Assiut University, Assiut, 71516 Egypt; University of Michigan-Shanghai Jiao Tong University Joint Institute, Shanghai, 200240 China

**Keywords:** Natural convection, Nanofluids, Thermophoresis, Inclined enclosure, Differentially heated enclosure, Bottom-heated enclosure, Two-phase lattice Boltzmann method

## Abstract

Laminar natural convection in differentially heated (*β* = 0°, where *β* is the inclination angle), inclined (*β* = 30° and 60°), and bottom-heated (*β* = 90°) square enclosures filled with a nanofluid is investigated, using a two-phase lattice Boltzmann simulation approach. The effects of the inclination angle on Nu number and convection heat transfer coefficient are studied. The effects of thermophoresis and Brownian forces which create a relative drift or slip velocity between the particles and the base fluid are included in the simulation. The effect of thermophoresis is considered using an accurate and quantitative formula proposed by the authors. Some of the existing results on natural convection are erroneous due to using wrong thermophoresis models or simply ignoring the effect. Here we show that thermophoresis has a considerable effect on heat transfer augmentation in laminar natural convection. Our non-homogenous modeling approach shows that heat transfer in nanofluids is a function of the inclination angle and Ra number. It also reveals some details of flow behavior which cannot be captured by single-phase models. The minimum heat transfer rate is associated with *β* = 90° (bottom-heated) and the maximum heat transfer rate occurs in an inclination angle which varies with the Ra number.

## Background

A nanofluid is a mixture of a small quantity of conducting nanoparticles suspended in a base fluid, such as water. A nanofluid is particularly known for its high, non-linear, and anomalous thermal conductivity, compared to the base fluid. Most recent studies also show an increase in heat transfer rate when a nanofluid is used in natural or forced convection in cavities, channels, etc. Despite the overwhelming number of publications, the heat transfer augmentation in several cases, such as natural convection in an enclosure utilizing a nanofluid, is not fully understood. Some of the results are contradictory to one another, due to several reasons, such as the lack of reliable experimental data and fundamental theoretical studies and accurate numerical simulations. While a nanofluid flow in general is a non-homogenous two-phase flow with a significant relative drift or slip velocity between particles and the base fluid, many works have assumed a homogenous mixture to simplify the simulation. This simplification may still provide a general picture and understanding of the problem, but in some cases, such as laminar natural convection, it may be a source of significant errors in estimating the Nu number and the convection heat transfer coefficient. In this paper, the state-of-the-art modeling approach, i.e., the lattice Boltzmann method (LBM) is employed. In recent years, the powerful LBM has been used to simulate heat transfer in nanofluids in various geometries, such as cavities and channels, e.g., [1–6]. When the LBM is used for nanofluids, the external forces acting on nanoparticles need to be considered, separately.

The important external forces that are responsible for creating a slip velocity on the surface of the suspended nanoparticles are discussed by Buongiorno [7]. In addition, the present authors [5, 6], among others, have shown that at certain conditions, slip velocity develops in natural convection in bottom-heated and differentially heated enclosures, as a result of external forces, such as thermophoresis and Brownian forces. These forces as well as the gravitational force play a significant role in the flow and heat transfer characteristics. This paper is an attempt to contribute to the physical understanding of the velocity slip mechanisms, and in particular the role of thermophoresis using a two-phase non-homogenous model. The thermophoresis role in nanofluids has been either neglected or in some works has been estimated inaccurately or erroneously. Some workers have used thermophoresis models that are only applicable to gases to model thermophoresis in liquids, causing errors as large as several orders of magnitude. The objective of this work is twofold: first, to use a two-phase lattice Boltzmann method that can model a slip velocity on particle surfaces which causes mixing and heat transfer augmentation and, second, to investigate the accurate contribution of thermophoresis as an external force and a mechanism that causes velocity slip and heat transfer augmentation.

Laminar and turbulent natural convection of nanofluids have been extensively studied in the bottom-heated and differentially heated enclosures. But very few works have been performed concerning inclined enclosures. Earlier studies on natural convection of pure fluids, such as air or water in enclosures with two parallel walls insulated and the other two walls kept at different temperatures, indicate that heat transfer rate in pure fluids changes with the inclination angle, where the lowest heat transfer rate occurs when the heated surface is on the top [8], if the enclosure rotation span is 360°. Similar observations are expected, when a nanofluid is used.

Some workers have used single-phase or homogenous models to study heat transfer and fluid flow in nanofluids in inclined and other geometries, e.g., [9–14]. In most cases, the external forces are neglected. In a differentially heated inclined enclosure, the heat transfer rate increases as the inclination angle increases up to an optimum inclination angle (45° for Ra = 10^4^, 30° for Ra = 10^5^ and 10^6^), beyond which the heat transfer rate decreases, e.g., [10, 12, 13]. Aminossadati and Ghasemi [11] studied heat transfer characteristics in an inclined square cavity with and without a central solid block. The inclination had no effect on heat transfer rate at low Ra numbers. At high Rayleigh numbers, with a central block, inclination enhanced heat transfer rate. This was not the case in the absence of the block.

The magneto-hydrodynamic (MHD) nanofluid in an inclined enclosure has been investigated as well [14]. The magnetic nature of the fluid changes the flow and heat transfer characteristics as compared to flows with conventional boundary conditions. In a mixed convection problem in an inclined channel and in the presence of a magnetic field, Noreen et al. [15] performed a numerical study where the effects of Brownian and thermophoresis forces on the mixed fluid flow in the channel were considered. Their analysis, however, was parametric assuming arbitrary Brownian and thermophoresis forces.

The problem of mixed natural and forced convection has been studied in an inclined enclosure, where the top and bottom walls were kept insulated but moving (lid-driven) [16]. This work was based on a single fluid, but a mixture model was used to capture the slip velocity. While the authors signified the importance of thermophoresis and Brownian motion on the slip velocity, these forces were not included in their simulation. Their results indicate that under the lid-driven boundary conditions, the addition of nanoparticles may change the flow from natural convection to forced convection. Fereidoon et al. [17] also studied the mixed convection in a lid-driven tilted enclosure, where the slip velocity due to Brownian and thermophoresis forces was neglected. In a recent work, MHD natural convection was studied in an inclined enclosure, where the effects of the strength and direction of the magnetic field on the flow and heat transfer characteristics were studied [18].

In most of the abovementioned works on natural convection in inclined enclosures, a single-phase homogenous model was used, and thermophoresis and Brownian forces were neglected or modeled inadequately [19], due to limitations in modeling of thermophoresis. The results in some cases are not consistent, and some fundamental physical phenomena are not adequately captured. Unfortunately, there is no experimental data on natural convection of nanofluids in inclined enclosures for model validation. Moreover, only recent experimental data on bottom-heated or differentially heated enclosures are reliable. The inaccuracy of some of the experimental data is due to nanoparticle agglomeration obscuring the effect of nanoparticle size and loading on heat transfer augmentation. Earlier experimental studies showed a decrease in heat transfer rate with the addition of nanoparticles to the base fluid, e.g., [20]. Recent experimental works [21, 22] are more reliable and consistent with the comprehensive theoretical analyses predicting heat transfer augmentation in nanofluids compared to the base fluid. It has been observed that the heat transfer rate increases up to a specific particle volume fraction.

While numerical simulations in nanofluids are abundant, fundamental understanding on the interaction between the nanoparticles and the base fluid and the effects of the external forces is limited. This is due to either using inadequate simulation techniques or neglecting some important effects, such as the external forces. In this paper, the state of the art in modeling a two-phase flow, i.e., the two-phase lattice Boltzmann method (LBM), is used for the simulation. Also, external forces are considered to capture the interaction between the phases. An accurate model for the estimation of thermophoresis force is used for the first time for natural convection in inclined enclosures. We study heat transfer and fluid flow in various inclination angles including *β* = 0° (a differentially heated enclosure), *β* = 30°, *β* = 60°, and *β* = 90° (bottom-heated).

## Methods

Although many earlier studies on nanofluids were performed using homogenous single-fluid models, i.e., assuming that the nanoparticles follow the base fluid streamlines, it is now commonly believed that the application of a single-phase homogenous model may not be adequate, particularly for natural convection [5, 6] or forced convection with low Re numbers [4]. When it comes to using a two-phase modeling approach, one may use the convectional transport equations for the base fluid, combined with a momentum equation for the nanoparticles as the dispersed phase. Alternatively, the multi-phase lattice Boltzmann method (LBM) may be employed. The LBM is used in this study, because from the microscopic point of view, the existing computational methods for conventional two-phase flows may fail to reveal the inherent nature of the flow and energy transport process inside a nanofluid. A microscopic or meso-scaled approach can better take into account the effect of interactions between the molecules and particles of the mixture. The lattice Boltzmann equation for a multicomponent flow is written as follows [23]:

1 $$ \begin{array}{l}{f}_i^{\sigma}\left(x+{e}_i\varDelta t,t+\varDelta t\right)-{f}_i^{\sigma}\left(x,t\right)=-\frac{1}{\tau^{\sigma }}\left({f}_i^{\sigma}\left(x,t\right)-{f}_i^{\sigma, \mathrm{eq}}\left(x,t\right)\right)+\left(\frac{2{\tau}^{\sigma }-1}{2{\tau}^{\sigma }}\right)\left(\frac{F_i^{\sigma }.{e}_i\varDelta t}{B_i{C}^2}\right)+\varDelta t{F}_i\ \\ {}\sigma = 1,\ 2\end{array} $$

where ${F}_i^{\sigma }$ represents the total inter-particle interaction forces and will be defined later (as *F*^*P*^, *F*^*w*^, Eqs. (23) and (24)). *F*_*i*_ is the natural convection driving force and is defined as follows:

2 $$ {F}_i={\omega}_i\rho g\beta \Delta t.\frac{e_i}{C_s^2} $$

In Eq. (1), *τ*^*σ*^ is the dimensionless collision-relaxation time constant for flow field of the *σ* component, *e*_*i*_ is the lattice velocity vector, the subscript *i* represents the lattice velocity direction, ${C}_s^2=\frac{C^2}{3},\ {f}_i^{\sigma}\;\left(x,\;t\right)$ is the density distribution function of the particles of component *σ* with velocity *e*_*i*_ at lattice position *x* and time *t*, and ${f}_i^{\sigma, \mathrm{eq}}\;\left(x,\;t\right)$ is the local equilibrium distribution function. One may select different forms of lattices, according to the hydrodynamic problem under study. For a two-dimensional problem, the well-known D2Q9 model is widely used. It is a nine-speed model based on a two-dimensional octagonal lattice in which the lattice velocity and parameter *B* in Eq. (1) are defined as follows [24]:

3 $$ {e}_i=\left(\begin{array}{c}\hfill \left(0,0\right)\kern4.32em i=0\hfill \\ {}\hfill C\left( \cos \frac{\left(i-1\right)\pi }{2}, \sin \frac{\left(i-1\right)\pi }{2}\right)\kern0.6em i=1,2,3,4\hfill \\ {}\hfill C\left( \cos \frac{\left(2i-9\right)\pi }{4}, \sin \frac{\left(2i-9\right)\pi }{4}\right)\kern0.24em i=5,6,7,8\hfill \end{array}\right. $$

4 $$ {B}_i=\left(\begin{array}{c}\hfill 0\kern2.52em i=0\hfill \\ {}\hfill \frac{1}{3}\kern2.52em i=1,2,3,4\hfill \\ {}\hfill \frac{1}{12}\kern2.28em i=5,6,7,8\hfill \end{array}\right. $$

where in Eq. (3), $C=\frac{\varDelta x}{\varDelta t}$ is the reference lattice velocity. The equilibrium distribution function ${f}_i^{\sigma, \mathrm{eq}}\;\left(x,\;t\right)$ should be carefully selected to ensure that each of the components obeys the macro-scaled Navier-Stokes equation. The particle distribution functions at the equilibrium state for a multicomponent flow could be written as follows [24]:

5 $$ {f}_i^{\sigma, \mathrm{eq}}={\omega}_i{\rho}^{\sigma}\left[1+\frac{3\;\left({e}_i.{u}^{\sigma, \mathrm{eq}}\right)}{C^2}+\frac{9\;{\left({e}_i.{u}^{\sigma, \mathrm{eq}}\right)}^2}{2{C}^4}+\frac{3\;{\left({u}^{\sigma, \mathrm{eq}}\right)}^2}{2{C}^2}\right] $$

where *ω*_*i*_, the weight coefficients, are written as follows:

6 $$ {\omega}_i=\left(\begin{array}{c}\hfill \frac{4\;}{9}\kern2.4em i=0\hfill \\ {}\hfill \frac{1}{9}\kern2.52em i=1,2,3,4\hfill \\ {}\hfill \frac{1}{36}\kern2.28em i=5,6,7,8\hfill \end{array}\right. $$

Similarly, one can introduce the lattice Boltzmann equation for the energy transport, by describing the energy equation as the energy distribution function ${g}_i^{\sigma }$. The lattice Boltzmann energy equation based on neglecting the viscous dissipation is written as follows [24]:

7 $$ \begin{array}{c}\hfill {g}_i^{\sigma}\left(x+{e}_i\Delta t,t+\Delta t\right)-{g}_i^{\sigma}\left(x,t\right)=-\frac{1}{\tau_{\theta}^{\sigma }}\left({g}_i^{\sigma}\left(x,t\right)-{g}_i^{\sigma, \mathrm{eq}}\left(x,t\right)\right)\;\hfill \\ {}\hfill \sigma =1,\;2\hfill \end{array} $$

where ${\tau}_{\theta}^{\sigma }$ is the dimensionless energy collision-relaxation time constant of the *σ* component. The energy distribution function at the equilibrium state is defined as follows:

8 $$ {g}_i^{\sigma, \mathrm{eq}}={\omega}_i{T}^{\sigma}\left[1+\frac{3\;\left({e}_i.{u}^{\sigma, \mathrm{eq}}\right)}{C^2}+\frac{9\;{\left({e}_i.{u}^{\sigma, \mathrm{eq}}\right)}^2}{2{C}^4}+\frac{3\;{\left({u}^{\sigma, \mathrm{eq}}\right)}^2}{2{C}^2}\right] $$

The macroscopic density, velocity, and temperature of each component are respectively calculated as follows:

9 $$ {\rho}^{\sigma }={\displaystyle \sum_{i=0}^{i=8}}{f}_i^{\sigma } $$

10 $$ {u}^{\sigma }={\displaystyle \sum_{i=0}^{i=8}}{f}_i^{\sigma }{e}_i $$

11 $$ {T}^{\sigma }={\displaystyle \sum_{i=0}^{i=8}}{g}_i^{\sigma } $$

By considering the internal and external forces, the macroscopic velocities and temperatures for nanoparticles and the base fluid are modified as follows [25–28]:

12 $$ {u}^{\sigma, \mathrm{eq}}=\frac{1}{\rho^{\sigma }}{\displaystyle \sum_{i=0}^{i=8}}{f}_i^{\sigma }{e}_i+\frac{F^{\sigma }{\tau}^{\sigma}\;\Delta t}{\rho^{\sigma }} $$

13 $$ {T}^{\sigma, \mathrm{eq}}={\displaystyle \sum_{i=0}^{i=8}}{g}_i^{\sigma }+\Delta t\;{\tau}_{\theta}^{\sigma}\frac{dT}{dt} $$

The kinematic viscosity *ν* and the thermal diffusivity *α* of the *σ* component are defined as follows:

14 $$ {\nu}^{\sigma }=\frac{2{\tau}^{\sigma }-1}{6}{C}^2\Delta t $$

15 $$ {\alpha}^{\sigma }=\frac{2{\tau}_{\theta}^{\sigma }-1}{6}{C}^2\Delta t $$

The average kinematic viscosity coefficient and thermal diffusivity of nanofluid are defined as follows:

16 $$ \nu =\frac{2{\displaystyle {\sum}_{\sigma }}{C}_{\sigma }{\tau}^{\sigma }-1}{6}{C}^2\Delta t $$

17 $$ \alpha =\frac{2{\displaystyle {\sum}_{\sigma }}{C}_{\sigma }{\tau}_{\theta}^{\sigma }-1}{6}{C}^2\Delta t $$

where

18 $$ {C}_{\sigma }=\frac{\rho^{\sigma }}{{\displaystyle {\sum}_{\sigma }}\rho } $$

In a laminar flow, turbulent eddies that potentially could cause a slip velocity due to an abrupt motion of the base fluid are absent, and therefore the other existing significant forces that may influence the suspended nanoparticles and cause a slip velocity include thermophoresis, Brownian, and gravitational forces [7, 29]. The thermophoresis force *F*_T_ may be related to the thermophoretic velocity *U*_T_ through the Stokes equation:

19 $$ {F}_{\mathrm{T}}=3\pi \mu {U}_{\mathrm{T}}{d}_{\mathrm{p}} $$

where *U*_T_ is related to the thermophoresis or thermodiffusion coefficient *D*_T_ as follows:

20 $$ {U}_{\mathrm{T}}=-{D}_{\mathrm{T}}\nabla T $$

Thermophoresis studies are more established and abundant in gases; however, theories developed for gases are not applicable to nanofluids, which are in liquid phase, as this leads to serious misinterpretation of the contribution of thermophoresis. Even the expression of MacNab-Meisen [30], which is prescribed for liquids, highly overestimates the thermophoresis strength, due to the presence of convection in their experiments. The most reliable expression that may be used for estimating thermophoresis coefficient in a liquid is based on the hydrodynamics theory and is used here [31, 32]:

21 $$ {D}_{\mathrm{T}}=2A\frac{k}{2k+{k}_{\mathrm{p}}}\frac{\mu }{\rho T} $$

where the physical properties are associated with the nanofluid (*k*, *μ*, *ρ*) and the particles (*k*_*p*_). Coefficient *A* is a function of the liquid physical properties and temperature [32]. For water, *A* is equal to 0.0085 [31]. The drag or friction force on small particles in a creeping flow is obtained from the Stokes law (Re < 1):

22 $$ {\mathbf{F}}_{\mathrm{D}}=3\pi {d}_{\mathrm{P}}\mu \left(\mathbf{V}-{\mathbf{V}}_{\mathrm{P}}\right) $$

Equation (22) provides a reasonable prediction for the drag force at the conditions of this study. Brownian motion is the random and fluctuating motion of particles caused by the collision of fluid molecules with the suspended nanoparticles. The components of the Brownian force are modeled as a Gaussian white noise process. Details of calculating the Brownian force exerted on particles in a fluid can be found elsewhere (e.g., [33] and references therein).

In the lattice Boltzmann method, the total force per unit volume acting on a nanoparticle of a single lattice is written as follows:

23 $$ {F}^{\mathrm{P}}=\frac{n}{V}\left[{F}_H+{F}_{\mathrm{D}}+{F}_{\mathrm{B}}+{F}_{\mathrm{T}}\right] $$

The force *F*_*H*_ is the sum of the buoyancy and weight forces. The sum of the forces per unit volume acting on the base fluid *F*^*w*^ is written as follows:

24 $$ {F}^w=-\frac{n}{V}\left[{F}_{\mathrm{D}}+{F}_{\mathrm{B}}+{F}_{\mathrm{T}}\right] $$

An empirical correlation was used for the viscosity of a nanofluid normalized by the viscosity of the base fluid [34]. The correlation by Maxwell is used for the effective thermal conductivity of a nanofluid. Water is used as the base fluid and 10-nm CuO nanoparticles as the dispersed phase with density of 6500 kg/m^3^ and specific heat capacity of 535.6 J/kg·K. Thermal conductivity of copper oxide (CuO) nanoparticles is variable; here an average value of 20 W/m·K is used. Although water is used as the base fluid, its thermal expansion coefficient is changed artificially to obtain a desired Ra number.

To test the mesh independence of the solution scheme for the given configuration used here, i.e., a 1.0 × 1.0 cm square cavity, four numerical experiments were performed with the mesh sizes of 100 × 100, 150 × 150, 200 × 200, and 300 × 300 at Ra = 10^6^, and *ϕ* = 0.0. It was found that the mesh size of 150 × 150 was accurate enough to interpret the flow and heat transfer in the cavity, with a reasonable amount of the computation time. The criteria for the convergence of the numerical solution for the flow and temperature fields are defined as follows:

25 $$ \left\Vert {\varepsilon}_1^{\sigma}\right.{\left.\left(i,j\right)\right\Vert}_{\infty}\le {10}^{-7},\;\mathrm{and}\;\left\Vert {\varepsilon}_2\right.{\left.\left(i,j\right)\right\Vert}_{\infty}\le {10}^{-7} $$

where

26a $$ {\varepsilon}_1^{\sigma}\left(i,j\right)=\frac{\sqrt{{\left({u}_x^{\sigma}\left(i,j,t+\Delta t\right)-{u}_x^{\sigma}\left(i,j,t\right)\right)}^2+{\left({u}_y^{\sigma}\left(i,j,t+\Delta t\right)-{u}_y^{\sigma}\left(i,j,t\right)\right)}^2}}{\sqrt{\left({u}_x^{\sigma }{\left(i,j,t+\Delta t\right)}^2+{u}_y^{\sigma }{\left(i,j,t+\Delta t\right)}^2\right)}} $$

26b $$ {\varepsilon}_{{}_2}^{\sigma}\left(i,j\right)=\frac{\mathrm{Abs}\ \left({T}^{\sigma}\left(i,j,t+\varDelta t\right)-{T}^{\sigma}\left(i,j,t\right)\right)}{\mathrm{Abs}\left({T}^{\sigma}\left(i,j,t+\varDelta t\right)\right)} $$

**Fig. 1:**
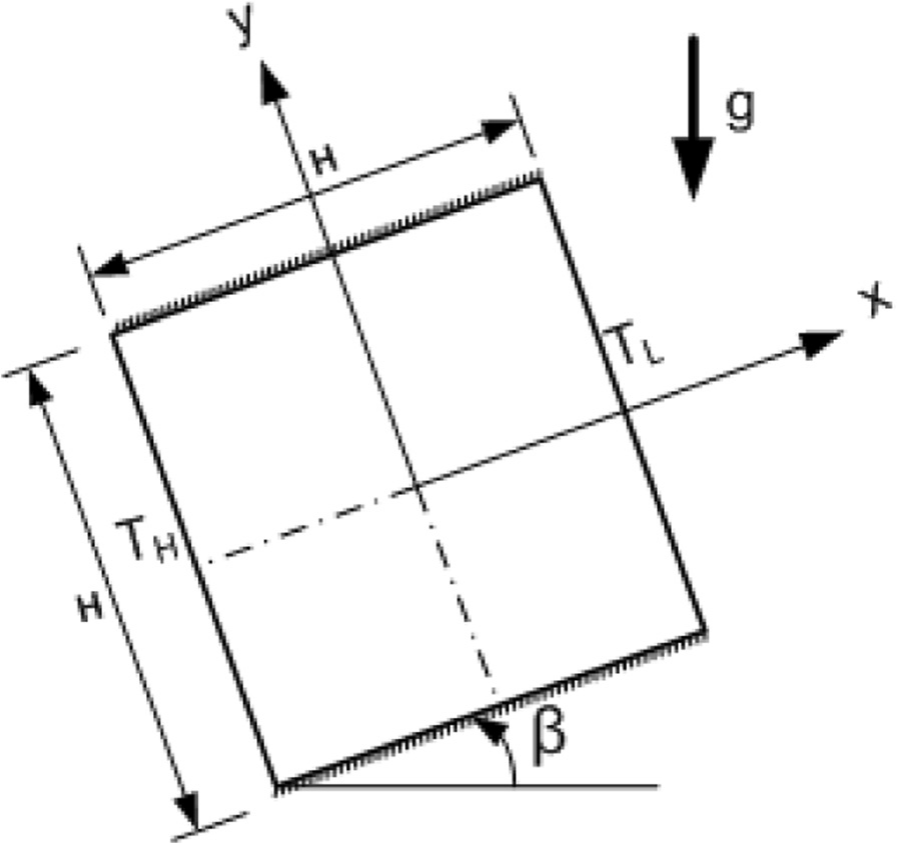
Schematic of the physical domain with the thermal boundary conditions and the coordinate system. All flow velocities at the walls are zero

A differentially heated enclosure is considered as the starting position of the cell (inclination angle *β* = 0), where the left wall is kept at a higher temperature *T*_H_, the right wall at *T*_L_, and the bottom and top walls are kept insulated. When the enclosure is tilted 90°, the bottom-heated configuration is retrieved. Intermediate angles of 30° and 60° are studied as well. Fig. 1 shows the problem geometry and boundary conditions. This is a thermal boundary driven flow with all velocities zero on the walls.

## Results and Discussion

**Fig. 2:**
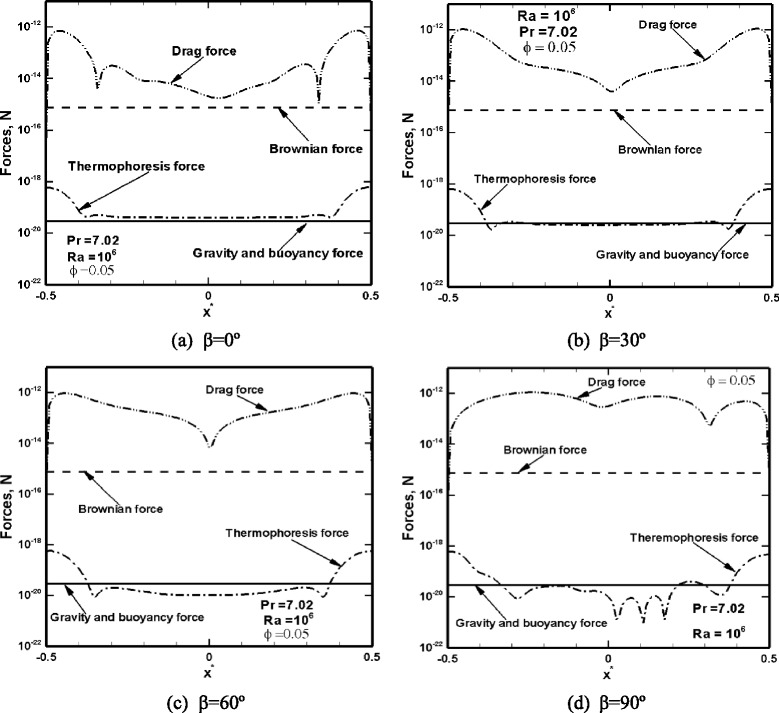
Variation of the resultant forces acting on a 10-nm nanoparticle along the *y/H* = 0.0 plane at *ϕ* = 0.05, Pr = 7.02, and Ra = 10^6^ for **a** *β* = 0°, **b** *β* = 30°, **c** *β* = 60°, and **d** *β* = 90°. The resultant forces are defined as follows: ${F}_{\mathrm{T}}=\sqrt{F_{\mathrm{T}x}^2+{F}_{\mathrm{T}y}^2},\ {F}_B=\sqrt{F_{\mathrm{B}x}^2+{F}_{\mathrm{B}y}^2},\ {F}_{\mathrm{D}}=\sqrt{F_{\mathrm{D}x}^2+{F}_{\mathrm{D}y}^2},\ {F}_H=\sqrt{{\left[\left({w}_{\mathrm{p}}-{F}_b\right) \cos \beta \right]}^2+{\left[\left({w}_p-{F}_b\right) \sin \beta \right]}^2}$

**Fig. 3:**
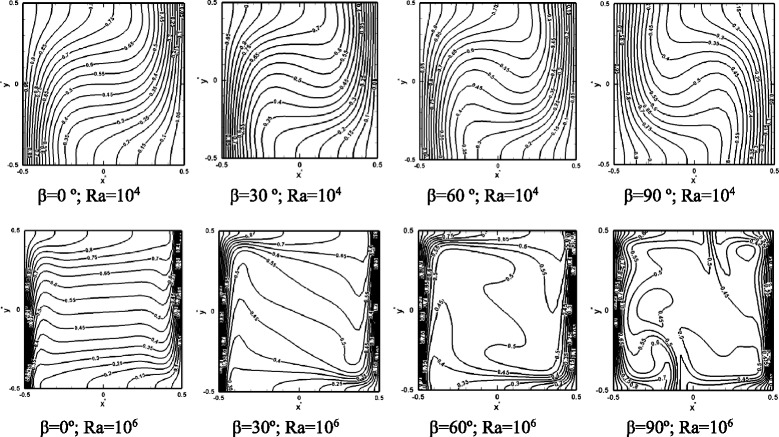
Isotherms at Ra = 10^4^ and Ra = 10^6^ at various inclination angles. Pr = 7.02 and *ϕ* = 0.05

We start presenting the results with investigation of the relative magnitude of the resultant gravitational, thermophoresis, Brownian, and drag forces applied on a 10-nm CuO particle at various inclination angles and Ra numbers. In all simulations, the direction of the gravitational acceleration remains downward; however, the components of each force are determined along the *x* and *y* directions, which may vary as *β* varies. Fig. 2 shows the resultant forces exerting on a 10-nm nanoparticle along the cavity, for various inclination angles, at a particle loading of 5 % and Ra = 10^6^. The resultant gravitational forces remain unchanged, although their *x* and *y* components change. The Brownian force is a function of the fluid temperature and particle size and therefore does not change with a change in the inclination angle. Thermophoresis force significantly changes due to alteration in the shape of isotherms, and thus the local temperature gradients (shown in Fig. 3). The drag force is an induced force and is created as a result of the existence of a slip velocity on the particle surface. Therefore, it is inferred that the drag force changes as a result of a change in slip velocity, and the slip velocity is created by Brownian, gravitational, and thermophoresis forces. The smallest forces acting on a 10-nm particle are the gravitational (weight and buoyancy) and thermophoresis forces with an order of magnitude of 10^−20^ N. Brownian force is of the order of 10^−15^ N, and the induced drag force, which is the largest force, is of the order of 10^−12^ to 10^−14^ N. Since the drag force exerted on particles varies with inclination angle, one may expect a change in the heat transfer rate, as well, as the inclination angle changes.

It was argued that a change in the thermophoresis force is due to a change in the local temperature gradients. To investigate this hypothesis, Fig. 3 shows the variation of the isotherms versus the inclination angle for a particle loading of 5 % (*ϕ* = 0.05). Considerable distortion in isotherms is observed with a change in the inclination angle. Similar qualitative observations of isotherms have been already made in previous works based on a single-phase model, e.g., [10]; however, the present results are more accurate given that a two-phase method is employed combined with a reliable expression for the thermophoresis force. At Ra = 10^4^, weak flows are induced in the cell, and therefore a gradual change from conduction limit at low Ra numbers to convection at higher Ra numbers is observed. Close to the hot and cold walls, the isotherms are parallel to the wall surface indicating a 1D conduction dominant heat transfer, while in the center of the cell, mixing and convection is stronger resulting in distortion of the isotherms. It is generally observed that at tilted angles of 30° and 60°, close to the hot and cold walls, the isotherms spacing decreases, indicating a higher heat transfer rate. As the Ra number increases, the spacing between the isotherms adjacent to the heated and cooled walls further decreases, which is indicative of an increase in the heat transfer rate. Also with an increase in the inclination angle, the isotherms at Ra = 10^6^ attain a more random contour form. The presence of the suspended nanoparticles and a relative drift or slip velocity between the particles and the base fluid is responsible for the irregular shape of the isotherms.

**Fig. 4:**
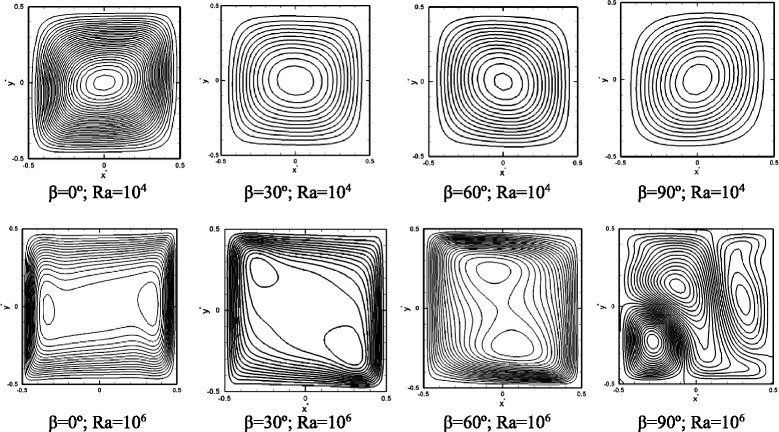
Flow streamlines at Ra = 10^4^ and Ra = 10^6^ at various inclination angles. Pr = 7.02 and *ϕ* = 0.05

Flow streamlines for various inclination angles are shown in Fig. 4 for a nanofluid with 5 % particle volume fraction. At Ra = 10^4^, where the induced flow is weak, the effect of the inclination angle is very weak, and in all cases, symmetric concentric single-cell circulations form, consistent with other pure and nanofluid works at low Ra numbers. It is noted that, in these cases, single-fluid models that assume a homogenous flow, also successfully predict the streamlines [9, 10, 12]. At Ra = 10^6^, however, at some inclination angles, particularly at 90° (bottom-heated), predictions of our two-phase flow model are different from refs. [9, 10, 12]. Depending on the magnitude of the Ra number and the extent of the flow perturbation, various solutions may be obtained for a bottom-heated enclosure (*β* = 90°) filled with a pure fluid; these solutions include a single-cell circulation, double-cell horizontal or vertical circulations, clockwise or counterclockwise [6, 35]. The presence of nanoparticles in our study may be the reason for obtaining a solution other than those for the standard pure fluid single-phase solutions, outlined in ref. [35]. When a single-phase model is used to find the streamlines in a bottom-heated cell at high Ra numbers (10^6^), the presence of nanoparticles makes the fluid more viscous and stable and a single-cell solution is obtained, similar to that reported in ref. [10]. Therefore, one may conclude that, only in some cases, a single-phase model may provide a qualitative solution for the flow patterns in a nanofluid.

**Fig. 5:**
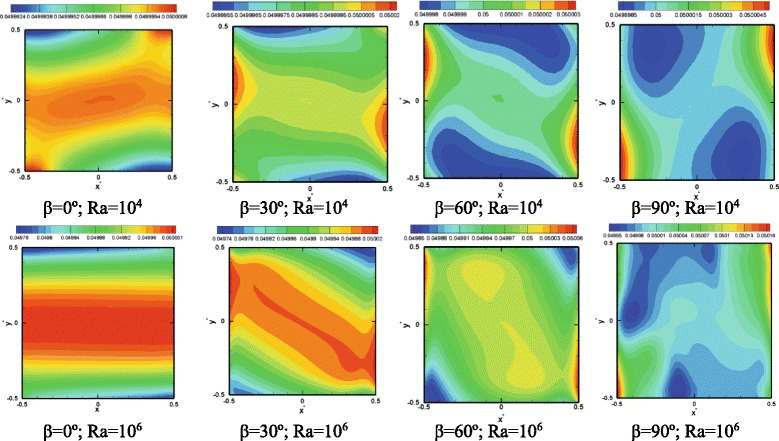
Nanoparticle volume fraction distribution at steady state for various inclination angles for Ra = 10^4^ and Ra = 10^6^. Pr = 7.02 and *ϕ* = 0.05

An interesting parameter is the distribution of nanoparticles within the cell at steady state. While the particles are initially evenly distributed within the cell, as a result of the existing forces and the fluid flow, particles may be redistributed, settled, or entrapped in the circulations. Figure 5 shows the distribution of the nanoparticle volume fraction at various inclination angles for particle volume fraction of 5 %. Overall, particles remain suspended with minimal accumulation. At Ra = 10^4^, particles are more uniformly distributed than for the case with Ra = 10^6^. Studies on micrometer-sized particle-laden flow usually show particle separation from the base fluid, e.g., [36].

**Fig. 6:**
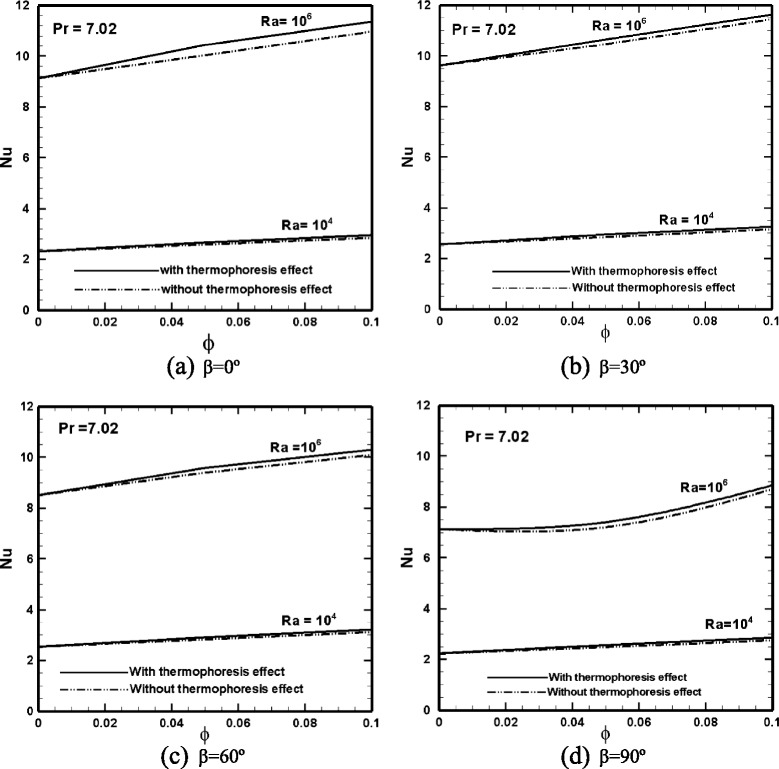
Variation of the average Nu number along the hot wall of the enclosure versus nanoparticle volume fraction at **a** *β* = 0°, **b** *β* = 30°, **c** *β* = 60°, and **d** *β* = 90°

In Fig. 6, the variation of the average Nu number on the hot wall [5] versus the particle volume fraction is displayed for various inclination angles and Ra numbers. The Nu number is significantly larger at Ra = 10^6^ compared to that at Ra = 10^4^, due to the development of a convective flow at Ra = 10^6^. Inclusion of nanoparticles has a higher effect on heat transfer enhancement at Ra = 10^6^, where a sufficiently strong convective flow has developed in the cell. At Ra = 10^4^, heat transfer is more dominated by conduction and the addition of nanoparticles has a weaker effect on heat transfer augmentation. In most cases, the Nu number increases linearly with an increase in the particle volume fraction. Note that the effect of considering thermophoresis in the simulations is also shown on the plots, where a significant heat transfer augmentation is observed at Ra = 10^6^, whereas at Ra = 10^4^, this effect is negated. Effect of the inclination angle on thermophoresis contribution can be also inferred. Thermophoresis has a larger contribution in heat transfer enhancement at *β* = 0 (differentially heated enclosure), where the relative percentage of increase in Nu number is 5 % at particle volume concentration of 5 %.

**Fig. 7:**
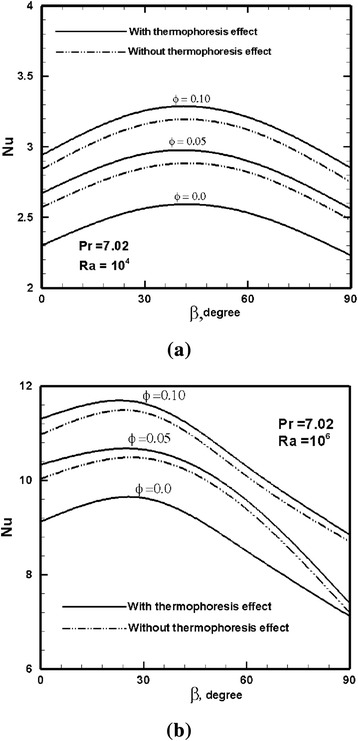
Variation of the average Nu number along the hot wall versus the inclination angle for various particle loadings for **a** Ra = 10^4^ and **b** Ra = 10^6^. Pr = 7.02

**Fig. 8:**
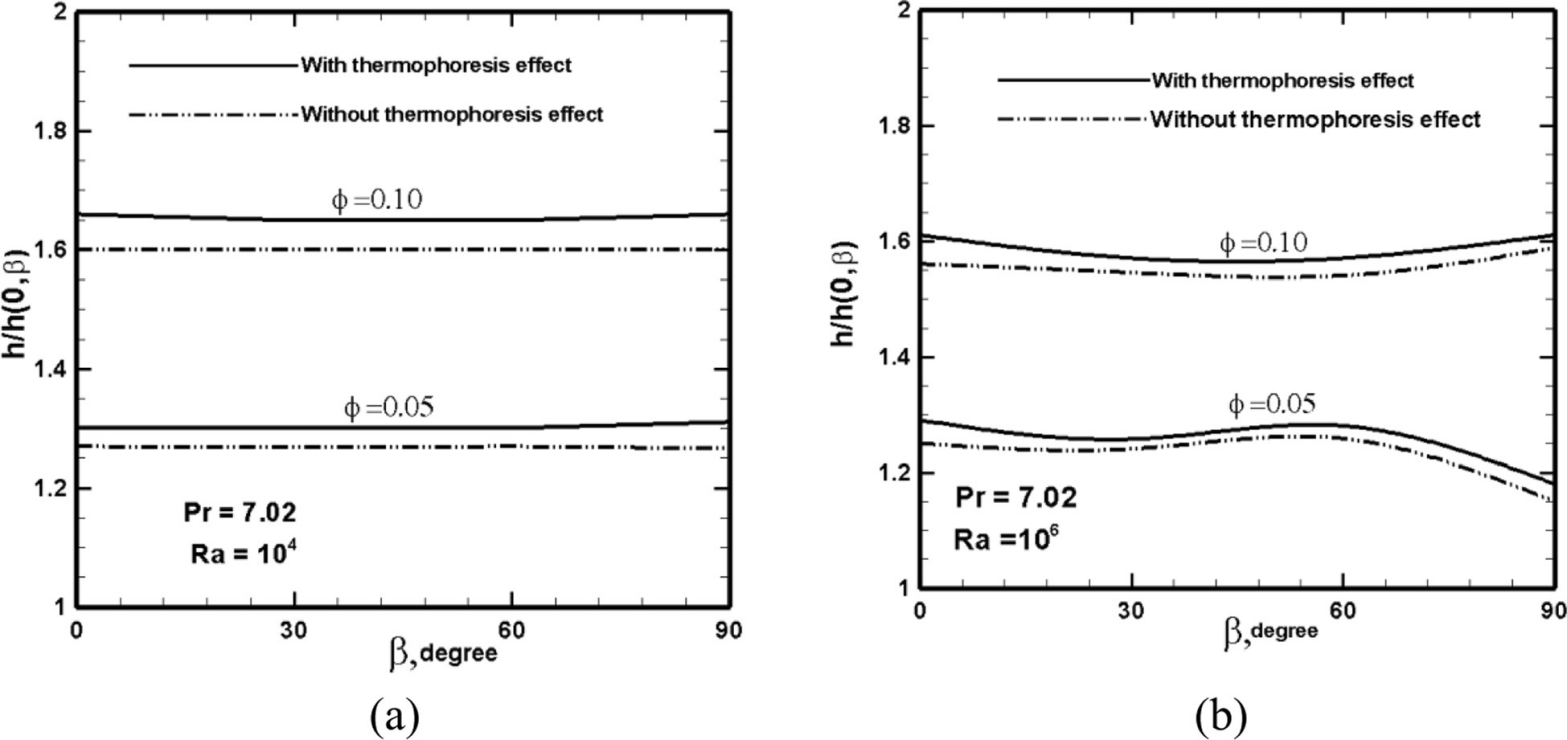
Variation of the normalized average heat transfer coefficient along the hot wall versus the inclination angle for various particle loadings at **a** Ra = 10^4^ and **b** Ra = 10^6^. Pr = 7.02. Note that *h* has been normalized with respect to *h*_0_ of the pure fluid at given *β*

Figures 7 and 8 investigate the effect of the inclination angle on the average Nu number, and normalized convection heat transfer coefficient with respect to the base fluid. The Nu number and convection heat transfer coefficients are averaged along the hot wall of the enclosure. The first observation is that these two nanofluid heat transfer parameters have a different behavior with respect to the inclination angle. The Nu number significantly changes with the inclination angle and particle loading, where it attains a maximum value at an inclination angle which is a function of the Ra number, and a local minimum value that corresponds to the bottom heating case, albeit within the range of the angles studied here. This has been observed by others, e.g., [10, 12], while the results of ref. [9] do not comply with these findings. We believe that the results presented here are more accurate owing to the two-phase flow LBM approach and proper modeling of the thermophoresis force. At Ra = 10^4^, the maximum Nu number occurs at about 40° (estimated as 45° in refs. [10, 12]), and at Ra = 10^6^, Nu number attains its maximum value at 26° (estimated as 30° in refs. [10, 12]). The contribution of thermophoresis force is also shown in Figs. 7 and 8.

The normalized heat transfer coefficient (*h*/*h*_0,*β*_) is shown in Fig. 8, where it is found that particularly at Ra = 10^4^, the normalized heat transfer coefficient is insensitive to the variation of the inclination angle. This indicates that the inclination angle has a similar effect on the heat transfer rate of both pure fluid (*h*_0,*β*_) and nanofluid (*h*). The increase in heat transfer coefficient of the nanofluid at two particle loadings can be readily inferred from this figure. At Ra = 10^4^, a 30 % increase in the convection heat transfer coefficient is observed with respect to the base fluid, and this is independent of the inclination angle. At Ra = 10^6^, a similar observation is made except that at angles larger than about 60° and at particle concentration of 5 %, *h*/*h*_0,*β*_ starts to decline, indicating that the positive effect of nanoparticle inclusion is mitigated at large angles. A similar effect is observed in Fig. 7b. We currently have no explanation for the occurrence of this effect at *ϕ* = 5 %.

## Conclusions

Laminar natural convection was studied in a heated square enclosure filled with a nanofluid covering the differentially heated, inclined and bottom-heated configurations. Motivated by the absence of accurate and multi-phase numerical simulations, the two-phase lattice Boltzmann method was employed where the interaction between the base fluid and the dispersion phase and also Brownian and thermophoresis forces were taken into consideration. Major findings are summarized as follows:

1. Application of a single-fluid homogenous model for natural convection of nanofluids may provide an approximate solution and a qualitative picture of the flow patterns. However, obtaining more accurate solutions and detailed physical insight requires a multi-phase model.
2. The contribution of thermophoresis on heat transfer augmentation was studied here using an expression that can fairly estimate the thermophoresis coefficient in nanofluids. Thermophoresis was found to have a considerable increasing effect in heat transfer rate of nanofluids of up to several percent.
3. Tilting the enclosure significantly alters the heat transfer rate in natural convection, and the effect is similar for both a pure fluid and a nanofluid. There is an angle associated with a maximum Nu number on the hot wall of the enclosure, which is a function of the Ra number and is independent of the particle volume concentration. At Ra = 10^4^, the maximum Nu number occurs at about 40°, and at Ra = 10^6^, the Nu number attains its maximum value at 26°, where 0° denotes a differentially heated enclosure.

## Nomenclature

*A* a coefficient in Eq. (21)

Abs absolute value

*c* nanofluid specific heat

$C=\frac{\varDelta x}{\varDelta t}$ reference lattice velocity

*C*_*σ*_ defined by Eq. (18)

*C*_*s*_ coefficient in Eq. (2)

*d*_P_ particle diameter

*D*_T_ thermodiffusion (thermophoresis) coefficient

*e*_*i*_ lattice velocity vector

${f}_i^{\sigma}\;\left(x,\;t\right)$ density distribution function of the particles of component *σ* at lattice position *x* along the direction *i*, and at time *t*

${f}_i^{\sigma,\;eq}\;\left(x,\;t\right)$ the local equilibrium distribution function of the particles of component *σ* at lattice position *x* along the direction *i*, and time *t*

${F}_i^{\sigma }$ total inter-particle interaction forces

*F*_*i*_ driving force for natural convection

*F*_b_ buoyancy force

*F*_B_ Brownian force

*F*_D_ drag force

*F*_*H*_ net gravitational force as a result of buoyancy and particle weight

*F*^P^ sum of forces acting on a particle per unit volume

*F*_T_ thermophoresis force

*F*^*w*^ sum of forces acting on the base fluid

*g* gravitational acceleration

${g}_i^{\sigma }$ energy distribution function

*h* convection heat transfer coefficient of nanofluid

*h*_0*,β*_ convection heat transfer coefficient of pure fluid at a given inclination angle

*H* dimension of the square cavity in *x* and *y* directions

*i* lattice velocity direction

*j* index of spatial coordinate in the *y* direction

*k* nanofluid thermal conductivity

*n* number of particles in a given lattice

Pr Prandtl number

Ra Rayleigh number

Re Reynolds number

*T*_H_ temperature of the hot wall

*T*_L_ temperature of the cold wall

*U*_T_ thermophoresis velocity

**V** flow velocity vector

**V**_**P**_ velocity vector of a particle

*w*_p_ particle weight

*x x* coordinate

*y y* coordinate

*t* time

Greek Symbols

*α* thermal diffusivity

*β* inclination angle

Δ*t* time step

*ε* convergence criterion

*ρ* nanofluid overall density

*ϕ* particle volume fraction/particle loading

*σ* components (*σ* = 1, 2, water and nanoparticles)

*μ* nanofluid viscosity

*τ*^*σ*^ dimensionless collision-relaxation time constant for the flow field of the *σ* component

${\tau}_{\theta}^{\sigma }$ dimensionless collision-relaxation time constant for the temperature field of the *σ* component

*θ* dimensionless temperature

*ν* kinematic viscosity

*ω*_*i*_ weight coefficients in Eq. (6)

Subscripts/Superscripts

eq equilibrium

*i* the lattice velocity direction
